# Protective Effects of Bu-Shen-Huo-Xue Formula against 5/6 Nephrectomy-Induced Chronic Renal Failure in Rats

**DOI:** 10.1155/2014/589846

**Published:** 2014-04-29

**Authors:** Jian-Rao Lu, Hai-Yan Han, Jie Chen, Chong-Xiang Xiong, Xin-Hua Wang, Jing Hu, Xiu-Feng Chen, Li Ma

**Affiliations:** ^1^Division of Nephrology, Shanghai Seventh People's Hospital, Shanghai 200137, China; ^2^School of Traditional Chinese Medicine, Capital Medical University, Beijing 100069, China

## Abstract

Chronic renal failure (CRF) is a serious disease related to increasing incidence and prevalence as well as decline in quality of life. Bu-Shen-Huo-Xue formula (BSHX), one of traditional herbal formulations, has been clinically employed to treat CRF for decades, but the mechanisms involved have not been investigated. In the present study, we investigated the effects of BSHX on some closely related parameters in 5/6 nephrectomy CRF rats. Rats with CRF were divided into five groups, namely, one control group, one enalapril group, and three BSHX treatment groups (0.25, 0.5, and 1 g/kg·d). The rats subjected to sham operation were used as a normal control. After eight weeks of treatment, BSHX significantly decreased the levels of Scr and BUN, downregulated the mRNA expression levels of TGF-*β*_1_, CTGF, NF-*κ*B, TNF-*α*, and OPN, upregulated the mRNA expression of PPAR*γ*, and reduced *in situ* expression of fibronectin and laminins. Histological findings also showed significant amelioration of the damaged renal tissue. BSHX protects 5/6 nephrectomy rats against chronic renal failure probably via regulating the expression of TNF-*α*, NF-*κ*B, TGF-*β*_1_, CTGF, PPAR*γ*, OPN, fibronectin, and laminins and is useful for therapy of CRF.

## 1. Introduction

The progression of chronic renal failure (CRF) is characterized by the development of glomerular and tubular lesions in which multiple factors can be involved [1]. The prevalence and incidence of the common disorder chronic kidney disease (CKD, also termed in its later stage CRF) are on the increase in developed and developing countries, which impose a very expensive and rising demand on health-care systems already burdened by paucity of resources [2]. In China, Korea, and Japan, extensive experience and abundant clinical data in the treatment of CRF have been documented for traditional Chinese medicine, which has been developed over thousands of years [3, 4]. Meanwhile, there have not been any effective treatments to hold back or treat CRF due to unclear pathogenesis. Therefore, there is a great need for the development of a therapeutic drug more efficient than the existing medication.

Bu-Shen-Huo-Xue formula (BSHX), a traditional Chinese medical formulation, is composed of seven medicinal plants, that is,* Astragalus membranaceus* (Fisch.) var.* mongholicus *(Bge.) Hsiao (Astragali radix),* Trigonella foenum-graecum* L. (Trigonellae semen),* Rheum palmatum* L. (Rhei radix et rhizoma),* Vaccaria segetalis* (Neck.) Garcke. (Vaccariae semen),* Curcuma phaeocaulis* Val. (Curcumae rhizoma),* Smilax glabra* Roxb. (Smilacis glabrae rhizoma), and* Coptis chinensis* Franch. (Coptidis rhizoma). BSHX has been clinically used to treat CRF in China, which possesses the efficacies of tonifying the kidney and nourishing qi and promoting blood circulation to remove blockage of channels. In clinical practice, BSHX significantly decreases the levels of serum creatinine (Scr) and blood urea nitrogen (BUN) and delays CRF progression [5, 6]. However, the mechanism involved has not been elucidated. Accordingly, we observed the effects of BSHX on several growth factors and cytokines in 5/6 nephrectomy rats with CRF, including transforming growth factor-*β*_1_ (TGF-*β*_1_), connective tissue growth factor (CTGF), tumor necrosis factor-*α* (TNF-*α*), nuclear factor kappa B (NF-*κ*B), peroxisome proliferator-activated receptor *γ* (PPAR*γ*), and osteopontin. Other related parameters, including Scr, BUN, fibronectin (FN), and laminins (LN), were also investigated. Our study results demonstrate that BSHX can reverse CRF by regulating the expression or activities of these parameters.

## 2. Materials and Methods

### 2.1. Preparation of the Extract

**Figure 1:**
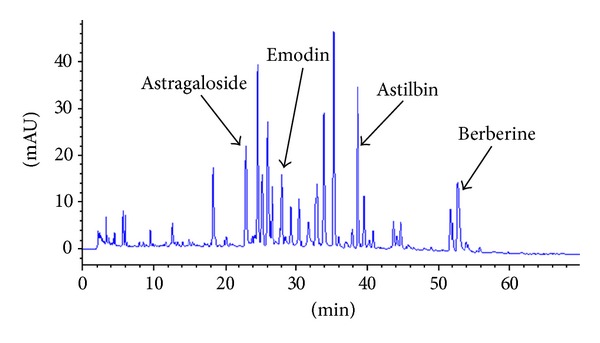
Fingerprint chromatogram of BSHX extract. Equipment type: Agilent1200; chromatographic column: Agilent Zorbax C18 (5 *μ*m, 250 × 4.6 mm); flow rate: 1 mL/min; column temperature: 30°C; wavelength of detection: 320 nm; liquid phase conditions: the proportion of acetonitrile/0.2% formic acid was from 5%/95% to 40%/60% within detection times of 0–70 min.

*Astragalus membranaceus* (Fisch.) var.* mongholicus *(Bge.) Hsiao (Astragali radix),* Trigonella foenum-graecum* L. (Trigonellae semen),* Rheum palmatum *L. (Rhei radix et rhizoma),* Vaccaria segetalis* (Neck.) Garcke. (Vaccariae semen),* Curcuma phaeocaulis* Val. (Curcumae rhizoma), Smilax glabra Roxb. (Smilacis glabrae rhizoma), and Coptis chinensis Franch. (Coptidis rhizoma) were purchased from Tongrentang Drugstore (Shanghai, China). The above Chinese traditional medicines (500 g) mixed in the proportion of 10 : 5 : 5 : 10 : 5 : 10 : 2 were pulverized in a motor-driven grinder to prepare the extract. After refluxing extraction with 5 L aqueous two times for 1 h each time, the extract was filtered and heat-dried at 50°C for* in vivo *evaluation. The yield was 13.7%. Fingerprint analysis by HPLC showed that numerous compounds were detected from the extract, such as astragaloside, emodin, astilbin, and berberine (Figure 1).

### 2.2. Animals and 5/6 Nephrectomy

Male Sprague-Dawley (SD) rats (175–185 g) were obtained from Shanghai Sippr BK Laboratory Animals Ltd. (Shanghai, China) and maintained under a 12 h light/12 h dark cycle, with food and water* ad libitum*. After 1 week, 5/6 nephrectomy was performed in rats under anesthesia with sodium pentobarbital (50 mg/kg^−1^ body weight, i.p.) by ablation of approximately 2/3 of the left kidney and then removal of the right kidney by ligation of renal artery, vein, and ureter ten days later. As an adaptive response to the renal ablation, the remnant kidney underwent hypertrophy, resulting in proteinuria, azotemia, and renal fibrosis with glomerular sclerosis and tubulointerstitial scarring over time [7].

### 2.3. Groups and Treatment

After two weeks of the operation, animals were divided into five groups, namely, one control group, one positive group, and three treatment groups, with twelve animals in each group, avoiding any intergroup differences in the levels of serum creatinine (Scr) and blood urea nitrogen (BUN). The rats undergoing a sham operation were used as normal control (sham group). Three treatment groups of rats were orally administered 0.25, 0.5, and 1.0 g/kg·d BSHX extract, respectively, by intubation. The positive group of rats received enalapril (10 mg/kg·d). The same volume of distilled water was given to the sham and control groups of rats. Eight weeks after successive treatment, the rats were sacrificed and blood samples were obtained. The renal tissue was quickly frozen and kept at −80°C until analysis.

### 2.4. Assays for BUN and Scr

The levels of Scr and BUN were measured using a Beckman Cx4 analyser (Fullerton, CA, USA), according to the description by He et al. [8].

### 2.5. RNA Isolation and Fluorescent Quantitative Reverse Transcription PCR (FQ-RT-PCR)

Total RNA of the renal tissue was extracted by homogenization in TRIzol (TRIzol box, from GIBCOBRC Inc., Shanghai, China). All of the isolated RNA samples were treated with the RNase-free DNase I (GIBCO BRC Inc., Shanghai, China) before FQ-RT-PCR. Reverse transcription was performed using a cDNA synthesis kit in accordance with the manufacturer's instructions (Applied Biosystems).

Primer pairs for rat genes (PPAR*γ*, TNF-*α*, NF-*κ*B, TGF-*β*, CTGF, and osteopontin) were designed using the Primer Express design software (Applied Biosystems) and listed in Table 1. The housekeeping gene GAPDH was used as an internal control. FQ-RT-PCR was performed on a real-time PCR instrument (ABI 7900HT, Applied Biosystems) and the relative expression of mRNA (%) was calculated according to the description by Zhang et al. [9].

### 2.6. Immunohistochemistry

Immunostaining of fibronectin and laminins in renal tissue was performed using the streptavidin-biotinylated peroxidase complex (SABC) method [10]. The positively stained area of the sections was analyzed with a computer-assisted pathological image analysis system (MPIAS-500, Shanghai, China).

### 2.7. Histological Study

A portion of the kidney tissue was cut, fixed with 10% buffered formalin, and embedded in paraffin for light microscopy. 3 *μ*m sections were stained with haematoxylin and eosin stain and the extent of glomerulus and tubulointerstitial damage was estimated.

### 2.8. Statistical Analysis

All results were presented as the mean ± SD. Data were analyzed using SPSS 13.0 statistical package. Data for multiple comparisons were performed by one-way ANOVA followed by Dunnett's test. A value of *P* < 0.05 was considered statistically significant.

## 3. Results

### 3.1. Effects of BSHX on BUN and Scr

**Figure 2:**
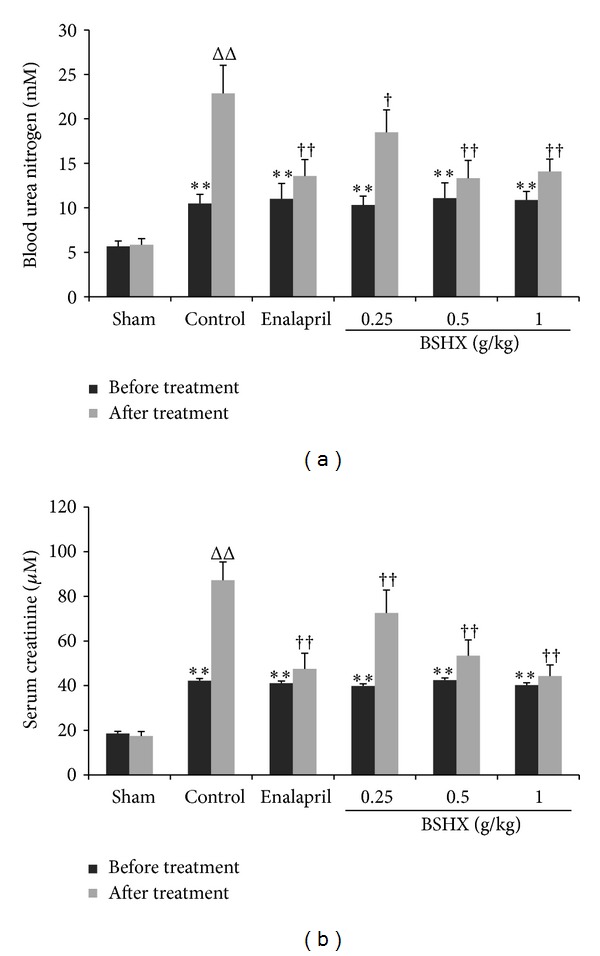
BSHX reduces the levels of BUN and Scr in 5/6 nephrectomy rats. After 5/6 nephrectomy rats were treated with BSHX at the doses of 0.25, 0.5, and 1.0 g/kg·d for eight successive weeks, BUN (a) and Scr (b) were analyzed. ^∗∗ΔΔ^ *P* < 0.01 compared with the sham group. ^†^ *P* < 0.05; ^††^ *P* < 0.01 compared with the control group. Data are expressed as the mean ± SD. *n* = 12.

Figure 2 shows the effects of BSHX on renal functional parameters. Before treatment, there were significant differences in the levels of BUN (a) and Scr (b) between the operation groups and the sham group but not any differences among the operation groups. When compared with the sham group, the levels of BUN and Scr were dramatically increased in the control group after eight weeks of distilled water treatment. However, BUN and Scr levels were evidently decreased by eight weeks of BSHX treatment (0.25, 0.5, and 1.0 g/kg) in comparison with the control group. Furthermore, there exists a dose-dependent relationship among the BSHX treatment groups on the Scr level (b).

### 3.2. Effects of BSHX on mRNA Expression of TGF-*β*_1_ and CTGF

**Figure 3:**
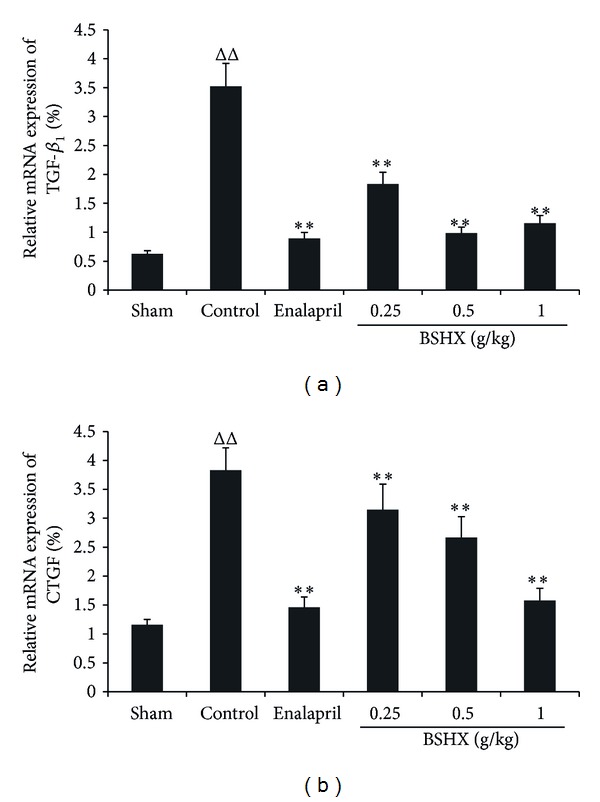
BSHX downregulates the mRNA expression of TGF-*β*_1_ and CTGF in the renal tissue of 5/6 nephrectomy rats. Following eight weeks of treatment with BSHX at the doses of 0.25, 0.5, and 1.0 g/kg·d, the mRNA expression of TGF-*β*_1_ (a) and CTGF (b) in the renal tissue was analyzed by FQ-RT-PCR. ^ΔΔ^ *P* < 0.01 compared with the sham group. **P < 0.01 compared with the control group. Data are expressed as the mean ± SD. *n* = 6.

There were very high mRNA expression levels of TGF-*β*_1_ (Figure 3(a)) and CTGF (Figure 3(b)) in both glomerular and tubulointerstitial injuries of rats when compared with the sham group. However, BSXH treatment for eight consecutive weeks evidently decreased their expression in comparison with the control group. TGF-*β*_1_ declined more significantly, but the dose-dependent reduction occurred in CTGF. There were no differences in the expression of TGF-*β*_1_ and CTGF between the high dose group and the enalapril group.

### 3.3. Effects of BSHX on mRNA Expression of PPAR*γ*, NF-*κ*B, and TNF-*α*

**Figure 4:**
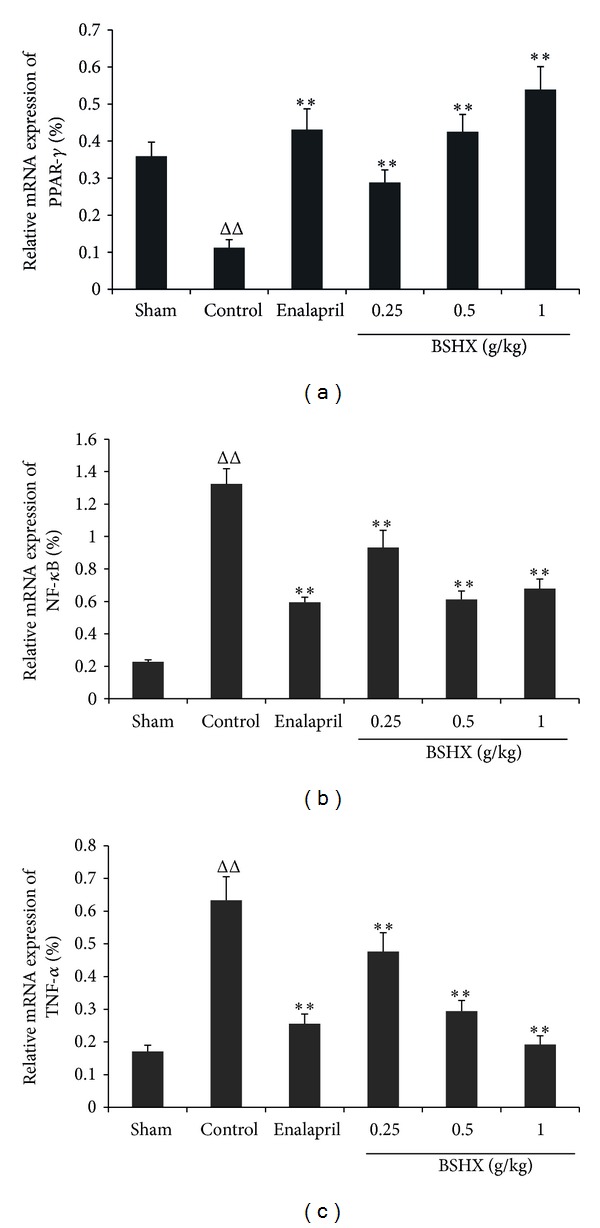
Effects of BSHX on mRNA expression of PPAR*γ*, NF-*κ*B, and TNF-*α* in renal tissue of 5/6 nephrectomy rats. After treatment with BSHX at the doses of 0.25, 0.5, and 1.0 g/kg·d for eight successive weeks, the mRNA expression of PPAR*γ* (a), NF-*κ*B (b), and TNF-*α* (c) in the renal tissue was analyzed by FQ-RT-PCR. ^ΔΔ^ *P* < 0.01 compared with the sham group. **P < 0.01 compared with the control group. Data are expressed as the mean ± SD. *n* = 6.

Figure 4 shows that there was an obvious decrease in PPAR*γ* but a significant increase in NF-*κ*B and TNF-*α* in the control group of rats when compared with the sham group of rats. However, PPAR*γ* was elevated and TNF-*α* was decreased evidently and dose-dependently by eight consecutive weeks of BSHX treatment. The mRNA expression of NF-*κ*B was also suppressed markedly but not dose-dependently.

### 3.4. Effects of BSHX on Osteopontin

**Figure 5:**
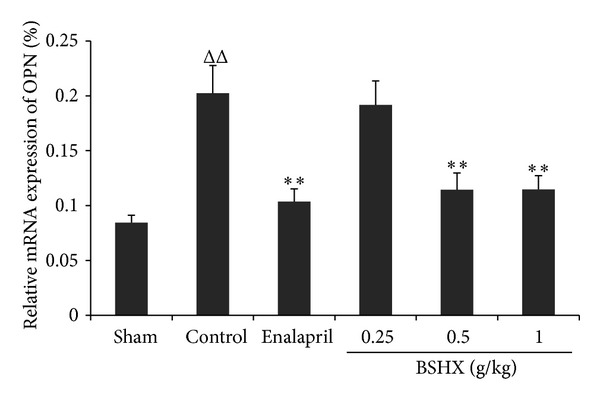
BSHX suppresses the mRNA expression of osteopontin in renal tissue of 5/6 nephrectomy rats. After 5/6 nephrectomy rats were treated with BSHX at the doses of 0.25, 0.5, and 1.0 g/kg·d for eight successive weeks, the mRNA expression of osteopontin in the renal tissue was analyzed by FQ-RT-PCR. ^ΔΔ^ *P* < 0.01 compared with the sham group. **P < 0.01 compared with the control group. Data are expressed as the mean ± SD. *n* = 6.

As shown in Figure 5, the mRNA expression level of osteopontin obviously rose in the control group of rats when compared with the sham group but was markedly downregulated by eight consecutive weeks of BSHX treatment at the doses of 0.5 and 1 g/kg·d. There were no differences revealed between the low dose group (0.25 g/kg·d) and the control group.

### 3.5. Immunohistochemical Findings

**Figure 6:**
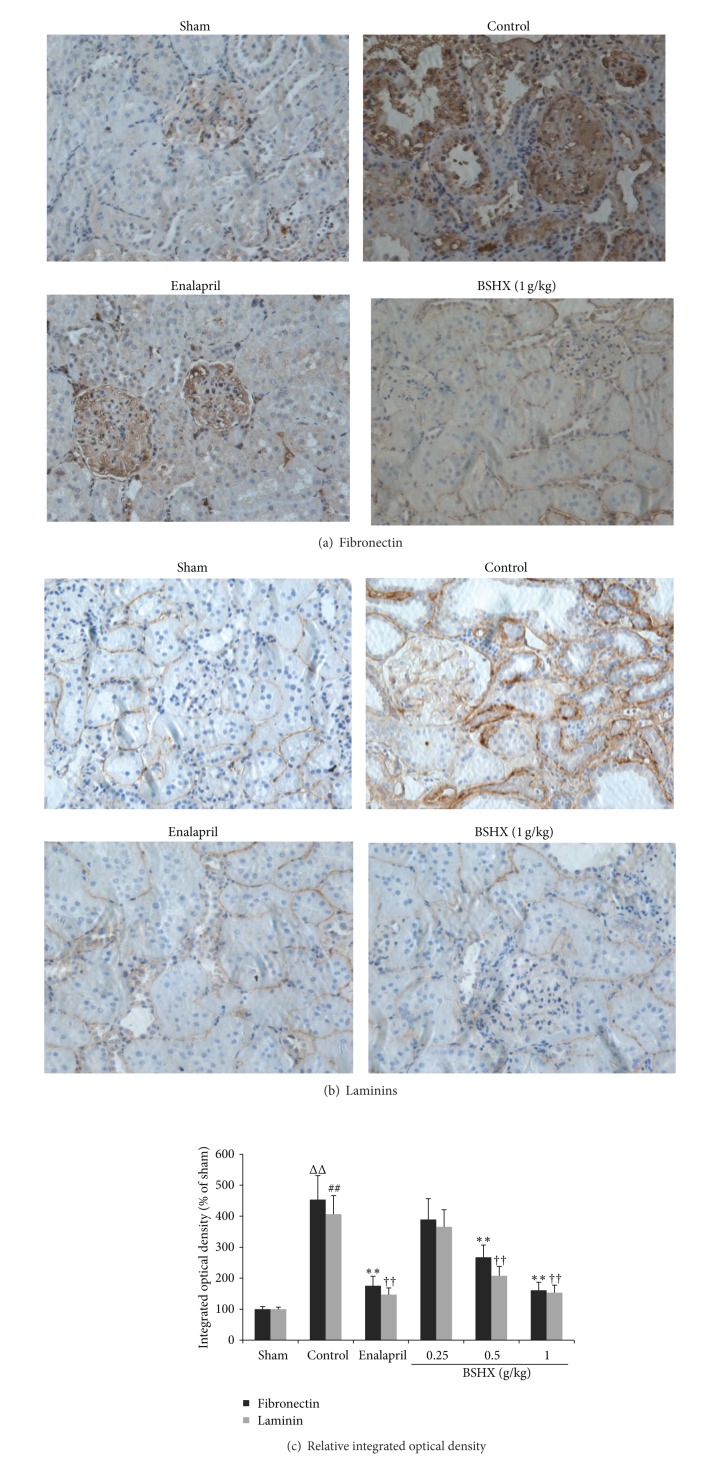
Immunohistochemical findings. (a) Fibronectin. (b) Laminins. (c) Relative integrated optical density. Immunohistochemical reactivities of fibronectin and laminins were found in the renal tissue of 5/6 nephrectomy rats. More areas and higher densities of fibronectin and laminins were shown in the control group, but the treatment group given the dose of 1 g/kg·d displayed fewer areas and lower densities than the control group. Figures for groups given BSHX at doses of 0.25 and 0.5 g/kg·d were not shown. Original magnification ×400. Relative integrated optical density was analyzed. ^ΔΔ##^ *P* < 0.01 compared with the sham group. ^∗∗††^ *P* < 0.01 compared with the control group. Data are expressed as the mean ± SD. *n* = 12.

Fibronectin (FN, Figure 6(a)) and laminins (LN, Figure 6(b)) are linearly distributed in glomerular basement membrane and tubular basement membrane in the renal tissue of normal rats. However, there were significantly increased expression levels of FN and LN distributed in glomerular basement membrane, renal interstitium, and tubular basement membrane in the control group of the renal tissue. Nevertheless, the raised levels of these proteins were significantly reversed by eight successive weeks of BSHX treatment although not restored back to normal.  Figure 6(c) reveals that BSHX treatment at the doses of 0.5 and 1 g/kg·d reduced the expression levels of FN and LN in the renal tissue of 5/6 nephrectomy rats markedly and dose-dependently. But the low dose of BSHX (0.25 g/kg·d) did not downregulate the expression levels of FN and LN when compared with the control group.

### 3.6. Histological Findings

**Figure 7:**
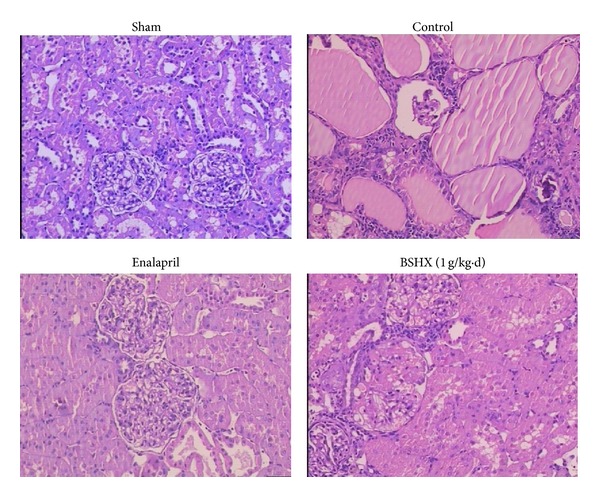
Histological characters of renal tissue sections. 5/6 nephrectomy elicited typical features of CRF tissue in rats. However, these changes were evidently attenuated by BSHX treatment for eight successive weeks. Figures for groups given BSHX at doses of 0.25 and 0.5 g/kg·d were not shown. Original magnification ×100.

Histological examination further confirmed the renal dysfunction in 5/6 nephrectomy animals (Figure 7). Figure 6(b) shows typical features of the CRF tissue compared with the sham group. These features included disordered glomerular structure, thick glomerular basement membrane, diffuse glomerular sclerosis and fibrosis, severe renal mesentery hyperplasia, obvious renal tubular dilation and fibrosis, interstitial edema, and a large amount of inflammatory cell infiltration. By contrast, these changes were evidently reversed by eight consecutive weeks of BSHX treatment.

## 4. Discussion

The 5/6 nephrectomy rat, a well-characterized model of chronic renal failure (CRF), features glomerulosclerosis and tubulointerstitial fibrosis, resulting in kidney dysfunction with significantly raised Scr and BUN. Scr reflects the ability of the kidney to remove creatinine from the blood and to concentrate it in the urine. The diseased or damaged kidney is less able to clear urea from the bloodstream, causing an elevated BUN [8]. In the current study, 5/6 nephrectomy rats showed sharp increases of Scr and BUN, which were evidently reversed by eight consecutive weeks of BSHX treatment, suggesting BSHX improves the kidney dysfunction of 5/6 nephrectomy rats.

Progressive CRF is the consequence of destructive tubulointerstitial fibrosis, glomerulosclerosis, and extracellular matrix (ECM) hyperplasia. Accumulation of ECM proteins is a conspicuous finding accompanying the progression of renal failure [8], which plays an important role in epithelial cell migration, differentiation, adhesion, and proliferation [11]. A number of cytokines mediate fibrosis by promoting cell proliferation, survival, and the deposition of ECM proteins.

Kidney inflammation may be followed by fibrosis and decline in renal function in the progressive CRF. The production of ECM proteins in the interstitium is induced and regulated by growth factors derived from macrophages and tubular cells. In these processes, transforming growth factor-*β*_1_ (TGF-*β*_1_) facilitates fibrosis and collagen synthesis in response to renal injury [8] and is one of alternate therapeutic targets for inhibition of fibrosis. Connective tissue growth factor (CTGF), a downstream mediator of TGF-*β*_1_, is positively related to TGF-*β*_1_ in promoting fibrosis, without mediating the immunosuppressive effects of TGF-*β*_1_. High mRNA expression of CTGF was associated with poor glomerular filtration rate (GFR) at baseline and subsequent deterioration of kidney function. CTGF can be generated* in vitro* in renal cells by a variety of stimuli, which induces fibroblast proliferation, ECM synthesis, and integrin expression, and may play significant roles in human renal diseases and fibrosis [12]. In the present study, the mRNA expression of TGF-*β*_1_ and CTGF was significantly increased in the control group but evidently reduced by eight consecutive weeks of BSHX administration. These results indicate that BSHX could reverse high mRNA expression of TGF-*β* and CTGF in the renal tissue of rats with CRF.

Tumor necrosis factor-*α* (TNF-*α*) is one of the most important inflammatory mediators secreted by macrophages [13]. The TNF-*α* expression was significantly different in the glomerulus and tubulointerstitium between the BSHX treatment groups and the control group, suggesting that BSHX alleviated inflammation in the renal tissue through preventing the expression of TNF-*α*. Peroxisome proliferator-activated receptor-*γ* (PPAR*γ*) belongs to the nuclear hormone superfamily of ligand-dependent transcription factors and has a significantly anti-inflammatory effect [14–16]. PPAR*γ* decreases the production of TNF-*α* not only by activation of anti-inflammatory gene but also by inhibition of the activity of proinflammatory transcription factors such as nuclear factor *κ*B (NF-*κ*B) [17]. In the present study, the mRNA expression of PPAR*γ* had a significant decrease in the kidney tissue of rats with CRF, but this change was significantly reversed by BSHX. Furthermore, BSHX also evidently suppressed NF-*κ*B mRNA expression in the renal tissue of rats with CRF. These results suggest that BSHX reduces TNF-*α* production via activation of PPAR*γ* to suppress NF-*κ*B activity.

Osteopontin (OPN), a secreted glycoprotein, was originally identified as a bone phosphoprotein secreted by the osteoid matrix. It binds tightly to hydroxyapatite, appearing to form an integral part of the mineralized matrix. It has been unclear whether increased OPN is the cause or the result of GFR decline [18]. Chronic kidney disease is characterized by progressive renal tissue fibrosis, and OPN may mediate the fibrotic process [19]. OPN is expressed at sites of calcification in human atherosclerotic plaques, emerging as an important inducible inhibitor of systemic calcification [18, 20], and may play a pivotal role in the progression of interstitial fibrosis in renal ischemia. However, the mechanism for OPN upregulation in kidney disease is not clear. In our study, the mRNA expression of OPN was significantly different in the renal tissue between the BSHX treatment groups and the control group. Overexpression of OPN was substantially suppressed by BSHX treatment in a dose-dependent manner, indicating one of the possible mechanisms for BSHX to improve renal function.

Fibronectin (FN) is a key ECM protein that has been shown to be upregulated in CRF. It is a glycoprotein of 250 kDa interacting with various matrix proteins and also modulates numerous cellular processes by interacting with cell surface receptors. Physiologically, FN plays an important role in cell adhesion, cell motility, and tissue repair [21]. Laminins (LN) are components of all basement membranes, specialized extracellular matrices found throughout the bodies of vertebrates and invertebrates, with diverse roles in fundamental developmental processes such as epiblast polarization and gastrulation, as well as in organ development and function [22, 23]. Laminins influence multiple functions of adjacent cells including adhesion, proliferation, and differentiation [24]. In this research, BSHX markedly decreased* in situ* expression of FN and LN, suggesting that BSHX inhibited FN and LN excessive aggregation in ECM.

In conclusion, BSHX reduces the levels of Scr and BUN, inhibits* in situ* expression of FN and LN, increases the mRNA expression of PPAR*γ*, and decreases the mRNA expression of TNF-*α*, NF-*κ*B, OPN, TGF-*β*_1_, and CTGF in the kidney tissue of rats with chronic renal failure. BSHX alleviates glomerulosclerosis and tubulointerstitial injury and subsequently reverses renal failure in 5/6 nephrectomy rats, possibly via inhibition of the inflammatory responses mediated by TNF-*α*, NF-*κ*B, TGF-*β*_1_, CTGF, PPAR*γ*, and OPN. The present study provides the evidence that BSHX can suppress gene expression associated with macrophage infiltration and renal fibrosis. BSHX is useful for slowing down CRF progression.

## Figures and Tables

**Table 1:** Primers used in FQ-RT-PCR analysis.

Gene	Primer sequence	Species	Amplicon size (bp)
PPAR-*γ*	Forward: 5′ CTGCTCCACACTATGAAGAC 3′	Rat	228
	Reverse: 5′ GGAAGCCTGATGCTTTATCC 3′		

TNF-*α*	Forward: 5′ TGGCGTGTTCATCCGTTC 3′	Rat	199
	Reverse: 5′ CTACTTCAGCGTCTCGTGTG 3′		

NF-*κ*B	Forward: 5′ TTACGGGAGATGTGAAGATGC 3′	Rat	103
	Reverse: 5′ TGAAGGTGGATGATGGCTAAG 3′		

TGF-*β*_1_	Forward: 5′ AAGGACCTGGGTTGGAAGTG 3′	Rat	125
	Reverse: 5′ TGGTTGTAGAGGGCAAGGAC 3′		

CTGF	Forward: 5′ ACCTACCGGGCTAAGTTC 3′	Rat	225
	Reverse: 5′ CTGGCTTTACGCCATGTC 3′		

Osteopontin	Forward: 5′ TCAATGGCTATGGACACC 3′	Rat	127
	Reverse: 5′ AGAAGGGACCTCCGAAAC 3′		

GAPDH	Forward: 5′ GTCGGTGTGAACGGATTTG 3′	Rat	181
	Reverse: 5′ TCCCATTCTCAGCCTTGAC 3′		
